# Copy number variation analysis in Chinese children with complete atrioventricular canal and single ventricle

**DOI:** 10.1186/s12920-021-01090-y

**Published:** 2021-10-09

**Authors:** Xingyu Zhang, Bo Wang, Guoling You, Ying Xiang, Qihua Fu, Yongguo Yu, Xiaoqing Zhang

**Affiliations:** 1 Department of Laboratory Medicine, Shanghai Children’s Medical Center, Shanghai Jiao Tong University School of Medicine, Shanghai, China; 2 Pediatric Translational Medicine Institute, Shanghai Children’s Medical Center, Shanghai Jiao Tong University School of Medicine, Shanghai, China; 3 Department of Pediatric Endocrinology and Genetic Metabolism, Shanghai Institute for Pediatric Research, Xinhua Hospital, Shanghai Jiao Tong University School of Medicine, Shanghai, China

**Keywords:** Copy number variations, Single ventricle, Complete atrioventricular canal, Congenital heart disease

## Abstract

**Background:**

Congenital heart disease (CHD) is one of the most common birth defects. Copy number variations (CNVs) have been proved to be important genetic factors that contribute to CHD. Here we screened genome-wide CNVs in Chinese children with complete atrioventricular canal (CAVC) and single ventricle (SV), since there were scarce researches dedicated to these two types of CHD.

**Methods:**

We screened CNVs in 262 sporadic CAVC cases and 259 sporadic SV cases respectively, using a customized SNP array. The detected CNVs were annotated and filtered using available databases.

**Results:**

Among 262 CAVC patients, we identified 6 potentially-causative CNVs in 43 individuals (16.41%, 43/262), including 2 syndrome-related CNVs (7q11.23 and 8q24.3 deletion). Surprisingly, 90.70% CAVC patients with detected CNVs (39/43) were found to carry duplications of 21q11.2–21q22.3, which were recognized as trisomy 21 (Down syndrome, DS). In CAVC with DS patients, the female to male ratio was 1.6:1.0 (24:15), and the rate of pulmonary hypertension (PH) was 41.03% (16/39). Additionally, 6 potentially-causative CNVs were identified in the SV patients (2.32%, 6/259), and none of them was trisomy 21. Most CNVs identified in our cohort were classified as rare (< 1%), occurring just once among CAVC or SV individuals except the 21q11.2–21q22.3 duplication (14.89%) in CAVC cohort.

**Conclusions:**

Our study identified 12 potentially-causative CNVs in 262 CAVC and 259 SV patients, representing the largest cohort of these two CHD types in Chinese population. The results provided strong correlation between CAVC and DS, which also showed sex difference and high incidence of PH. The presence of potentially-causative CNVs suggests the etiology of complex CHD is incredibly diverse, and CHD candidate genes remain to be discovered.

**Supplementary Information:**

The online version contains supplementary material available at 10.1186/s12920-021-01090-y.

## Background

Congenital heart disease (CHD) is the most common birth defect with an incidence of 1–1.2% in live births [1, 2]. Due to disrupted early-stage development, CHD consists of many structural malformations of the cardiovascular system, ranging from simple lesions such as atrial septal defects (ASD) and ventricular septal defects (VSD), to complex lesions such as tetralogy of Fallot (ToF), complete atrioventricular canal (CAVC) and single ventricle (SV). Although clinical treatment have significantly improved, complex CHD still remains to be a leading cause of newborn-related mortality [3].

Consistent with the complexity of early heart development, the etiology of CHD is multifactorial. To date, only about 20–30% of CHD cases could be attributed to genetic or environmental causes based on available technologies [4–6]. The incidence of some specific CHD types has been revealed with sex or race biases [7, 8]. The recurrence risk of CHD in the offspring of an affected parent, as well as in the siblings of a CHD child, has been reported to be higher than the general population [9, 10]. The evidences emphasize that genetics plays an important role in the pathogenesis of CHD [11].

Both small and large genetic variations could contribute to CHD [12]. Small insertions and deletions (INDELs), ranging from 1 bp to 10 kb in length [13], are typically detected by sequencing technologies [14]. Putative deleterious small variants in single genes could cause both syndromic and isolated CHD. For instance, Noonan syndrome, a common genetic disorder, is mostly caused by mutations in *PTPN11* gene. Pulmonary valve stenosis and CAVC represent relatively common features in Noonan syndrome [15, 16]. Heterotaxy syndrome, which comprises a class of congenital disorders resulting from malformations in left–right body patterning, has been reported to be associated with mutations in *NODAL*, *ACVR2B*, *LEFTY2*, *GDF1*, *ZIC3*, *CRELD1* and *NKX2.5* [17]. Majority patients with heterotaxy syndrome have serious CHD including SV [17, 18]. In addition to syndromic CHD, an increasing number of genes have been identified in individuals with isolated CHD [11]. Whole exome sequencing and whole genome sequencing are able to effectively identify small variants associated with CHD.

Large genetic variants, including aneuploidies, chromosomal rearrangements and copy number variations (CNVs), are also important genetic causes of CHD. CNVs can range in size from single genes to large contiguous deletions or duplications of millions of base pairs [19, 20]. Pathogenic CNVs tend to be large, de novo and disrupting coding regions [20]. Although recent advances in next generation sequencing showed their potential in CNVs detection, chromosome microarray, either array comparative genomic hybridization or single nucleotide polymorphism array, is still the gold standard for CNV detection and validation [21].

Nowadays, investigation of genes in overlapping CNV regions can probably identify relevant genes or refined intervals for certain genetic diseases [22–24]. Considering the heterogeneity of CHD etiology, a large number of CNVs associated with CHD have been identified over the past decades, especially the conotruncal anomalies including TOF, TGA and pulmonary atresia (PA)/VSD [25–27]. In our study, genome-wide CNVs in Chinese children with CAVC and SV were screened since there were scarce researches dedicated to these two types of CHD.

## Methods

### Study subjects

We obtained a cohort of 528 children diagnosed as CAVC (n = 264) or SV (n = 264) by echocardiography from the Shanghai Children’s Medical Center between November 2010 and August 2019. The patients had an average age at 8.77 ± 2.77 (mean ± SD) years. The phenotypic details of this cohort were summarized in Additional file 1: Table S1. The Ethics Committee of the Shanghai Children’s Medical Center reviewed and approved this study (SCMCIRB-K2017009).

### DNA extraction

Genomic DNA was isolated from peripheral blood samples of all patients using Gentra Puregene Blood Kit (QIAGEN, Hilden, Germany) according to manufacturers’ instructions. NanoDrop2000 spectrophotometer (Thermo Fisher Scientific, Waltham, MA) was used to check the quantity and quality of the DNA samples. Only samples with OD260/OD280 ratios between 1.8 and 2.0, and OD260/OD230 ratios > 1.5 were selected for further investigation.

### Microarrays

Microarrays were designed based on the Affymetrix Arrays platforms (Thermo Fisher Scientific, Waltham, MA), namely the CytoScan 750 K arrays. We deleted probes with high population frequency and added probes particularly designed for sites marked with two stars in Clinvar as well as pathogenic variants in HGMD. In the meantime, design of probes was also based on clinical data of high-morbidity diseases in newborns, which was applied to screen for CHD, especially CHD patients accompanied by extra-cardiac anomalies. Genomic DNA samples were amplified, fragmented and stringently hybridized onto arrays according to manufacturers’ instructions. Microarrays were automatedly processed by GeneTitan Multi-Channel instruments together with Affymetrix Command Console (AGCC) Software for instruments control and production of probe cell intensity data (CEL file).

### Data analysis

Microarray data processing was implemented using the Affymetrix Chromosome Analysis Suite v2.0 (ChAS) Software, and CNVs were called based on human assembly GRCh38 (hg38). There were 521 patients including 262 CAVC and 259 SV passed the QC tests finally.

Only CNV calls larger than 200 kb and with at least 50 probes for deletion and duplication were considered for further analysis. The detected CNVs calls were identified by public databases and websites: Database of Genomic Variants (DGV, http://dgv.tcag.ca/dgv/app/home), Online Mendelian Inheritance in Man (OMIM, https://omim.org/), UCSC Genome Browser (https://genome.ucsc.edu/), the Clinical Genome Resource (ClinGen, https://www.clinicalgenome.org/), Database of Chromosomal Imbalance, PubMed (https://www.ncbi.nlm.nih.gov/pubmed/), Phenotype of Humans using Ensemble Resources (DECIPHER, http://decipher.sanger.ac.uk/), SCAN (http://www.scandb.org/newinterface/about.html), ClinVar (https://www.ncbi.nlm.nih.gov/clinvar/).

In this study, the detected CNVs were classified according to the following criteria: (1) The ones having ≥ 70% overlap with CNVs reported in DGV were categorized into non-causative CNVs. And the rest CNVs were identified as potentially-causative CNVs. The frequencies for non-causative CNVs and potentially-causative CNVs were calculated based on DGV and DECIPHER database, respectively. (2) In our cohort of 262 CAVC and 259 SV patients, the CNVs had a frequency less than 1% were defined as rare CNVs, and the others (≥ 1% in our dataset) were identified as common CNVs. (3) Novel CNVs were those have not been previously reported in the literature or available public database.

## Results

### Clinical characteristics of the 528 patients

In 264 CAVC patients, the sex ratio (Males to Females) was 0.98 (131:133) and the average age was 8.80 ± 3.76 (mean ± SD) years. In 264 SV patients, the sex ratio was 1.34 (151:113) and the average age was 7.04 ± 3.57 years. The results were summarized in Table 1.

**Table 1:** Clinical characteristics of the 528 patients

CHD classification	Sex	Numbers of children	Average age (Mean ± SD)	Total
CAVC	Female	133	8.80 ± 3.76	264
	Male	131		
SV	Female	113	7.04 ± 3.57	264
	Male	151		

CAVC, complete atrioventricular canal; SV, single ventricle

### CNV detection in CHD cases

In this study, seven samples failed the QC criteria (CAVC209, CAVC211, SV134, SV145, SV177, SV181, SV254). Among the rest 521 samples (262 CAVC and 259 SV) who passed the QC tests, a total of 3465 CNVs were detected with a median size of 922.3 kb (max 23.9 Mb, min 51.5 kb). Large CNVs with size greater than 200 kb were selected for further analysis.

Nine large CNVs (according to the filtering criteria) were identified in 44 CAVC cases (16.79%, 44/262), which consists of 4 duplication CNVs involved 406 genes and 5 deletion CNVs affecting 452 genes (see Table 2). We also identified 18 large CNVs in 16 SV cases (6.06%, 16/264), including 13 duplications and 5 deletions (see Table 3).

**Table 2:** CNVs in 262 CAVC patients

ID	Sex	Cardiac phenotype	CNV	Start (bp)	End (bp)	size (Kb)	Marker count	Chr, region	Frequency (%) in our cohort^#^	CNV Type	Frequency (%) in DGV*	Frequency (%) in DECIPHER*	DECIPHER/ISCA/OMIM	Major candidate genes
CAVC149	M	CAVC, DORV, TAPVC, SA	Dup	16,174,790	18,642,949	2468	104	Yq11.221–11.222	0.3817 (1/262)	N-causative	0.0545			
CAVC084	M	CAVC, ASD, AVVR	Del	6,492,863	9,264,427	2772	163	Yp11.2	0.3817 (1/262)	N-causative	0.0138			
CAVC142	M	CAVC, ASD, PFO	Del	42,673,287	42,903,979	231	122	17q21.2-17q21.31	0.3817 (1/262)	N-causative	N. A			
CAVC274	F	CAVC, PFO	Dup	99,471,505	100,319,058	848	70	3q12.1-3q12.2	0.3817 (1/262)	P-causative		0.0130 (5/38367)	VSD	*TBC1D23*
CAVC145	F	CAVC, PDA, PH	Del	141,845,636	144,390,430	2545	396	8q24.3	0.3817 (1/262)	P-causative		0.0417 (16/38367)	VSD, CAVC, ASD, ToF	*PUF60*
CAVC102	F	CAVC, PDA, PFO	Del	78,588,699	84,286,313	5698	373	8q21.13-8q21.2	0.3817 (1/262)	P-causative		0.0678 (26/38367)	TOF, VSD	*STMN2, ZBTB10*
CAVC162	M	CAVC, DORV	Del	73,230,400	74,752,194	1522	256	7q11.23	0.3817 (1/262)	P-causative		0.336 (129/38367)	CAVC	*ELN*
CAVC207	F	CAVC, ASD, AVVR	Dup	18,721	38,816,830	38,798	4451	9p24.3-9p13.1	0.3817 (1/262)	P-causative		0.8106 (311/38367)	TOF, TGA, CoA	*uncertain*
CAVC084	M	CAVC, ASD, AVVR	Dup	13,038,109	46,687,133	33,649	3449	21q11.2-21q22.3	14.8855 (39/262)	P-causative		0.5578 (214/38367)	ASD, VSD, ToF, HLHS	
CAVC142	M	CAVC, ASD, PFO	Dup	13,038,109	46,443,844	33,406	3433	21q11.2-21q22.3						
CAVC003	F	CAVC, TOF	Dup	13,038,109	46,687,133	33,649	3449	21q11.2-21q22.3						
CAVC006	M	CAVC, TOF, ASD	Dup	13,038,109	46,687,133	33,649	3449	21q11.2-21q22.3						
CAVC035	M	CAVC, ASD, PDA	Dup	13,038,109	46,687,133	33,649	3449	21q11.2-21q22.3						
CAVC050	M	CAVC, ASD, VSD	Dup	13,038,109	46,687,133	33,649	3449	21q11.2-21q22.3						
CAVC056	F	CAVC, PH	Dup	13,038,109	46,687,133	33,649	3449	21q11.2-21q22.3						
CAVC061	F	CAVC, ASD, PDA	Dup	13,038,109	46,687,133	33,649	3449	21q11.2-21q22.3						
CAVC083	F	CAVC, PDA, AVVR	Dup	13,038,109	46,687,133	33,649	3449	21q11.2-21q22.3						
CAVC085	F	CAVC, PFO, PH	Dup	13,038,109	46,687,133	33,649	3449	21q11.2-21q22.3						
CAVC088	F	CAVC, ASD, PH	Dup	13,038,109	46,687,133	33,649	3449	21q11.2-21q22.3						
CAVC115	F	CAVC, ASD, AVVR	Dup	13,038,109	46,687,133	33,649	3441	21q11.2-21q22.3						
CAVC117	F	CAVC, ASD, PS	Dup	13,038,109	46,687,133	33,649	3449	21q11.2-21q22.3						
CAVC120	F	CAVC, PFO, PH	Dup	13,038,109	46,687,133	33,649	3449	21q11.2-21q22.3						
CAVC137	F	CAVC, ASD, PDA	Dup	13,038,109	46,687,133	33,649	3449	21q11.2-21q22.3						
CAVC138	F	CAVC, PFO, AVVR	Dup	13,038,109	46,687,133	33,649	3449	21q11.2-21q22.3						
CAVC158	M	CAVC, PH, AVVR	Dup	13,038,109	46,687,133	33,649	3449	21q11.2-21q22.3						
CAVC164	M	CAVC, PFO, PH	Dup	13,038,109	46,687,133	33,649	3449	21q11.2-21q22.3						
CAVC181	F	CAVC, ASD, PH	Dup	13,038,109	46,687,133	33,649	3409	21q11.2-21q22.3						
CAVC182	F	CAVC, ASD, PH	Dup	13,038,109	46,687,133	33,649	3449	21q11.2-21q22.3						
CAVC215	F	CAVC, ASD, AVVR	Dup	13,038,109	43,656,492	30,618	2869	21q11.2-21q22.3						
CAVC225	M	CAVC, TOF, ASD	Dup	13,038,109	44,280,050	31,242	2942	21q11.2-21q22.3						
CAVC226	F	CAVC, ASD, AVVR	Dup	13,038,109	42,995,775	29,958	2772	21q11.2-21q22.3						
CAVC230	F	CAVC, PH, AVVR	Dup	13,038,109	43,025,182	29,987	2774	21q11.2-21q22.3						
CAVC232	M	CAVC, ASD, AVVR	Dup	13,038,109	42,687,189	29,649	2753	21q11.2-21q22.3						
CAVC237	F	CAVC, PH	Dup	13,038,109	43,055,843	30,018	2784	21q11.2-21q22.3						
CAVC241	M	CAVC, ASD, AVVR	Dup	13,038,109	44,068,398	31,030	2955	21q11.2-21q22.3						
CAVC248	F	CAVC, ASD, PH	Dup	13,038,109	46,687,133	33,649	3425	21q11.2-21q22.3						
CAVC250	F	CAVC, TOF, PDA	Dup	13,038,109	46,687,133	33,649	3449	21q11.2-21q22.3						
CAVC251	F	CAVC, ASD	Dup	13,038,109	46,687,133	33,649	3434	21q11.2-21q22.3						
CAVC252	M	CAVC, ASD, AVVR	Dup	13,038,109	46,687,133	33,649	3449	21q11.2-21q22.3						
CAVC253	M	CAVC, ASD, PH	Dup	13,038,109	44,282,471	31,244	2976	21q11.2-21q22.3						
CAVC259	M	CAVC, ASD, PH	Dup	13,038,109	45,989,774	32,952	3232	21q11.2-21q22.3						
CAVC260	F	CAVC, PDA, PS	Dup	13,038,109	43,638,875	30,601	2902	21q11.2-21q22.3						
CAVC264	F	CAVC, ASD, PH	Dup	13,301,510	44,117,172	30,816	2948	21q11.2-21q22.3						
CAVC268	M	CAVC, PDA, PFO	Dup	13,038,109	43,789,291	30,751	2895	21q11.2-21q22.3						
CAVC271	F	CAVC, ASD, PH	Dup	13,038,109	46,687,133	33,649	3449	21q11.2-21q22.3						
CAVC278	M	CAVC, PH	Dup	13,038,109	46,440,013	33,402	3431	21q11.2-21q22.3						
CAVC274	F	CAVC, PFO	Dup	13,038,109	46,687,133	33,649	3449	21q11.2-21q22.3						

Details of CNVs in 262 CAVC patients were presented, including 3 non-causative CNVs (N-causative) and 6 potentially-causative CNVs (P-causative). “Dup” means duplication; “Del” means deletion; “CN” means copy number; “N.A” means not available. # Frequencies in our cohort (Common, ≥ 1%; Rare, < 1%). * Frequencies referred to corresponding public dataset (DGV for non-causative CNVs, Decipher for potentially-causative CNVs). Potentially-causative CNV frequency in Decipher was calculated with specific CNV counts dividing by the number of open-access patient records

**Table 3:** CNVs in 259 SV cases

ID	Sex	Cardiac Phenotype	CNV	Start (bp)	End (bp)	size (Kb)	Marker Count	Chr,region	Frequency (%) in our cohort^#^	CNV Type	Frequency (%) in DGV*	Frequency (%) in DECIPHER*	DECIPHER/ISCA/OMIM	Major candidate genes
SV039	M	SV, CAVC, PS	Del	60,756,434	61,430,513	674	94	14q23.1	0.3861 (1/259)	N-causative	0.0176			
SV130	F	SV, SA, CAV	Dup	149,504,294	150,375,282	871	91	Xq28	0.3861 (1/259)	N-causative	0.8100			
SV136	M	SV, MGA, PS	Del	38,016,208	38,115,650	99	69	22q13.1	0.3861 (1/259)	N-causative	0.0046			
SV139	F	SV, CAVC, MGA, SA	Dup	215,435,747	216,250,781	815	73	2q35	0.3861 (1/259)	N-causative	N. A			
SV140	M	SV, ASD, MGA	Dup	109,713,114	110,674,457	961	111	2q13	0.3861 (1/259)	N-causative	0.0742			
SV143	M	SV, bilateral right-sideness isomerism, CAV, MGA, SA	Dup	120,423,759	121,529,013	1105	154	2q14.2	0.3861 (1/259)	N-causative	N. A			
SV168	F	SV, ASD, PDA, PA	Del	38,031,511	38,115,657	84	67	22q13.1	0.3861 (1/259)	N-causative	0.0046			
SV176	M	SV, VSD, SA, PS	Dup	30,969,138	31,111,965	143	79	7p14.3	0.3861 (1/259)	N-causative	0.0324			
SV220	M	SV, bilateral right-sideness isomerism, MGA, SA	Del	70,952,592	71,144,199	192	101	5q13.2	0.3861 (1/259)	N-causative	1.5418			
SV226	F	SV, bilateral right-sideness isomerism, MGA, SA, AVVR	Dup	127,821,735	128,377,797	556	89	5q23.2-5q23.3	0.3861 (1/259)	N-causative	N. A			
SV228	M	SV, DORV, CAVC, PFO	Dup	36,101,999	36,481,789	380	59	17q12	0.3861 (1/259)	N-causative	0.3247			
SV261	F	SV, CAVC, dextrocardia, PS, MGA, SA	Dup	10,789,416	11,070,136	281	131	Xp22.2	0.3861 (1/259)	N-causative	0.0046			
SV261	F	SV, CAVC, dextrocardia, PS, MGA, SA	Dup	40,813,257	41,931,182	1118	79	7p14.1	0.3861 (1/259)	P-causative		0.0104 (4/38367)	VSD	*INHBA*
SV143	M	SV, bilateral right-sideness isomerism, CAV, MGA, SA	Dup	167,970,535	169,617,000	1646	154	5q34-5q35.1	0.3861 (1/259)	P-causative		0.0391 (15/38367)	VSD, CoA	*TENM2, SLIT3*
SV147	F	SV, ASD, SA	Dup	100,407,113	101,349,032	942	136	Xq22.1	0.3861 (1/259)	P-causative		0.1277 (49/38367)	VSD	*PCDH19*
SV163	M	SV, TGA, CoA	Dup	225,487,757	227,180,965	1693	238	1q42.12-1q42.13	0.3861 (1/259)	P-causative		0.0547 (21/38367)	VSD	*LEFTY1, LEFTY2*
SV195	M	SV, mesocardia, PS, SA, MGA, atrial situs inversus	Dup	71,674,242	72,187,074	513	66	2p13.2	0.3861 (1/259)	P-causative		0.0078 (3/38367)	VSD	*CYP26B1*
SV007	M	SV, SA, PS	Del	80,259,789	88,874,526	8615	581	8q21.13-8q21.3	0.0860 (33/38367)	P-causative		0.0860 (33/38367)	TOF, VSD	*ZBTB10*

The details of CNVs in 259 SV patients were presented, including 12 non-causative CNVs (N-causative) and 6 potentially-causative CNVs (P-causative). “Dup” means duplication; “Del” means deletion; “CN” means copy number; “N.A” means not available. # Frequencies in our cohort (Common,  ≥ 1%; Rare, < 1%). * Frequencies referred to corresponding public dataset (DGV for non-causative CNVs, Decipher for potentially-causative CNVs). Potentially-causative CNV frequency in Decipher was calculated with specific CNV counts dividing by the number of open-access patient records

There were 15 non-causative CNVs identified, including 3 in CAVC samples (Yp11.2, 17q21.2-17q21.31, Yq11.221–11.222) and 12 in SV patients (14q23.1, Xq28, 22q13.1, 2q35, 2q13, 2q14.2, 22q13.1, 7p14.3, 5q13.2, 5q23.2-5q23.3, 17q12, Xp22.2). The rest CNVs were identified as potentially-causative CNVs. Most CNVs identified in our study were rare (< 1%), occurring just once among the CAVC (0.38%, 1/262) or SV samples (0.39%, 1/259) except the 21q11.2-21q22.3 duplication in CAVC cohort (14.89%, 39/262).

### Potentially-causative CNVs in CAVC cases

In 262 CAVC patients, 6 potentially-causative CNVs were identified in 43 cases (16.41%, 43/262) (see Table 2). Surprisingly, 90.70% CAVC patients with these CNVs (39/43) were found to carry duplication of 21q11.2–21q22.3 which was considered as a common CNV (14.89%, 39/262) in our cohort. Among them, there was one patient (CAVC274) simultaneously had a ~ 0.8 Mb duplication at 3q12.1–3q12.2 encompassing *COL8A1*, *HP09053*, *FILIP1L*, *CMSS1* and *TBC1D23*. For the deletion CNVs, we had identified isolated deletions of 7q11.23 (CAVC162), 8q21.13-8q21.2 (CAVC102) and 8q24.3 (CAVC145) separately.

Additionally, we also consulted the DECIPHER and ISCA databases for evidences of clinical relevance. Duplication of 9p24.3-9p13.11 (CAVC207) has been reported to associate with ToF/TGA/coarctation of the aorta (CoA) phenotype.

### Potentially-causative CNVs in SV cases

Totally, 6 cases with potentially-causative CNVs were identified in 259 SV patients (2.32%, 6/259) (see Table 3), and none of them were identified as trisomy 21. Except deletion of 8q21.13–8q21.3, the rest were all duplication CNVs (5q34-5q35.1, Xq22.1, 1q42.12-1q42.13, 2p13.2, 7p14.1). These CNVs have been reported to associate with VSD, ToF and CoA in DECIPHER and ISCA.

Additionally, we noticed that two patients were simultaneously identified with two CNVs. One patient (SV143) was identified with one non-causative CNV (2q14.2, dup) and one potentially-causative CNV (5q34–5q35.1, dup), and the other patient (SV261) owned one non-causative CNV (Xp22.2, dup) as well as one potentially-causative CNV (7p14.1, dup).

## Discussion

CAVC, accounts for ~ 4% of CHD, is a complex cardiac malformation characterized by a variable deficiency of the atrioventricular area in the developing heart [28, 29]. SV, one of the most common forms of severe CHD, comprises a spectrum of congenital cardiac malformations defined by severe underdevelopment of one ventricle [30].

In 262 CAVC patients reported here, 14.89% (39/262) carried the duplication of 21q11.2-21q22.3, which could be diagnosed as trisomy 21, namely Down Syndrome (DS). A striking association of CAVC with DS was found in this study. All DS patients had the same ~ 3.3 Mb duplication at 21q11.2-21q22.3, and a systematic reanalysis indicated that 21q22.13 was the minimal critical region to the DS phenotype [31]. Additionally, another study detected 57.6% cardiac malformations in 500 patients with DS, and it also suggested CAVC (35.1%) was the most frequent heart anomaly [32]. It is putative that CAVC is the most frequent type of CHD in DS patients, and our study also provide strong evidence for this correlation in Chinese population. Additionally, CAVC also referred to as complete atrioventricular septal defect, and it has been reported that AVSD (atrioventricular septal defects) are more common in the female of DS patients [33]. In our study, the female/male ratio of CAVC with DS patients was 1.60 (24:15), which suggest that potential sex differences existed in the prevalence of CAVC in DS patients. Besides, we also noticed that rates of pulmonary hypertension (PH) in DS patients with CAVC was 41% (16/39), which was higher than previous report (28%, 364/1242) [34]. In fact, it was well known that PH is common in children with DS, and our study intensely proved this correlation.

Nowadays, several genes located in the “CHD critical region” on chromosome 21 have been proved to be associated with CAVC, including *DSCAM*, *COL6A1*, *COL6A2*, and *DSCR1* [35]. However, there were three DS patients simultaneously had another CNV located at different chromosome in our cohort, and one of the CNVs (3q12.1–3q12.2 dup) has been reported to associate with VSD in Decipher database. Additionally, several DS patients showed not only CAVC (4/27), but also other cardiac anomalies, such as ToF, ASD, patent foramen oval (PFO) and patent ductus arteriosus (PDA). Although the above-mentioned genes can explain partial cardiac phenotypes in DS patients, the genetic causes still were difficult to clarify especially when DS probands accompanied with multiple CNVs and diverse CHD phenotypes.

In our study, two potentially-causative CNVs had been identified as main causes of certain syndromes with heart anomalies. The microdeletion on 7q11.23 caused Williams-Beuren Syndrome (WBS; OMIM 194050), which is a multisystemic developmental disorder mostly accompanied with CHD [36, 37]. More than 90% of WBS patients have the ~ 1.55 Mb pair deletion extending from *FKBP6* to *GTF2I*, and it has been widely accepted that the deletion or mutation of an *elastin* (*ELN*) allele is a major cause of WBS [38]. One patient in this study (CAVC162) had a ~ 1.52 Mb deletion at 7q11.23 extending from *NCF1B* to *GTF2I*, encompassing the *ELN* gene. The other microdeletion on 8q24.3 have been recognized as associated with Verheij syndrome (OMIM 615583), which is characterized by growth retardation, developmental delay (DD), microcephaly, vertebral anomalies, dysmorphic features, cardiac and renal defects [39]. Poly(U) Binding Splicing Factor 60 (*PUF60*) were suggested as the main cause for heart defects in the syndrome, since knockdown of *Puf60* alone resulted in cardiac structural defects [40]. The patient (CAVC145) reported here had a ~ 2.5 Mb deletion of 8q24.3, representing with growth retardation and heart anomalies.

Among the rest CNVs identified in the CAVC patients, CNVs located at 8q21.13–8q21.2, 9p24.3–9p13.1 and 3q12.1–3q12.2 have been seldom reported. The detected potentially-causative deletion CNV, 8q21.13–8q21.2, encompasses Zinc Finger and BTB Domain Containing 10 (*ZBTB10)*, which has been known as a CHD gene. *ZBTB10* encodes a telomere-associated protein [41]. Lately, a GWAS involving 4,000 unrelated Caucasian patients diagnosed with CHD indicated that *ZBTB10* was associated with TGA, since two highly significant SNPs (rs148563140 and rs143638934) closely located to this gene [42]. Furthermore, they suggested strong cell-type specificity in murine cardiac development for *Zbtb10*. Except the known CHD gene *ZBTB10*, this CNV region in the patient (CAVC102) also included *STMN2* related to abnormality of the cardiovascular system. *STMN2* encodes a member of the stathmin family of phosphoproteins, functioning in microtubule dynamics and signal transduction [43]. Compared with controls, methylation of *STMN2* significantly increased (FDR *p* value = 4.27 × 10^–51^) in VSD cases [44]. Besides, it has been shown that *Stmn2* expresses in atrioventricular node, endocardium and outflow tract in mouse according to the LifeMap Discovery database. For the remaining two duplication CNVs, one of them (9p24.3–9p13.1, CAVC207) has been reported as VSD or TOF in DECIPHER. In this region, only *Rfx3* gene was in “ventricular septal defect” derived from the MGI (mouse genome informatics) database. For the duplicated region of 3q12.1–3q12.2, a report had shown a VSD patient had a ~ 116 kb duplication of this region, and *TBC1D23* has been identified as the major candidate gene [25]. In our study, the patient (CAVC274) had a ~ 0.8 Mb duplication at 3q12.1–3q12.2, encompassing this CHD candidate gene *TBC1D23*.

There were 6 potentially-causative CNVs (8q21.13–8q21.3, 1q42.12–1q42.13, Xq22.1, 5q34–5q35.1, 2p13.2, 7p14.1) in 259 SV patients. The deleted region of 8q21.13-8q21.3 (SV007) overlapped with the above-mentioned CNVs in the CAVC patient (CAVC102). Furthermore, we noticed that duplication of 1q42.12–1q42.13 (SV163) included 2 known CHD risk genes, *LEFTY1* and *LEFTY2* [45]. Especially, It has been reported that the SNP rs2295418 in the *LEFTY2* gene is associated with CHD in Chinese Han populations [46]. For other detected CNVs, only several genes were included in the deleted or duplicated regions, namely Xq22.1 (SV147; *PCDH19*, *TNMD*), 2p13.1 (SV195; *DYSF*, *CYP26B1*, *EXOC6B*) and 7p14.1 (SV176, *INHBA* and *SUGCT*). There were no related reports between CHD and these detected CNVs, so we focused on three genes (*PCDH19*, *CYP26B1* and *INHBA*) with a significantly higher pLI score, which reflects the intolerance to the loss of function mutations. *PCDH19* (Protocadherin 19) is a member of the delta-2 protocadherin subclass of the cadherin superfamily. *CYP26B1* (cytochrome P450 family 26 subfamily B member 1) involves in limiting retinoic acid (RA) levels within vertebrate embryos, which facilitate RA degradation [47]. It has been well-known that RA is important for the development of the heart. *INHBA* (Inhibin Subunit Beta A) encodes a member of the TGF-beta (transforming growth factor-beta) superfamily of proteins, and it has been shown as a candidate gene for cardiac development [48].

As for the proband (SV143) with a ~ 1.6 Mb duplication at 5q34-5q35.1, we found that this region encompasses 2 genes (*SLIT3* and *TENM2*) related to septal defects of heart. *SLIT3* (Slit Guidance Ligand 3) expressed in cardiomyocyte-like progenitor cells [49], and membranous ventricular septum defects as well as atrioventricular and aortic valve abnormalities are exhibited in *SLIT3*-mutant mice [50]. Recently, S*LIT3* variants in humans has shown association with CHD involving in ToF and septal and outflow tract defects [51]. *TENM2* (Teneurin Transmembrane Protein 2) expresses abundantly in human fetal heart. Moreover, patients with loss of *TENM2* presented ASD in Decipher database, gain of *TENM2* didn’t show any phenotype of CHD yet.

Structural genetic changes, especially copy number variants, represent a major source of genetic variation contributing to CHD patients. In recent years, a large number of CNVs associated with CHD have been identified [25–27]. Nevertheless, the role of pathogenic CNVs in SV and CAVC remain largely unknown because of their low incidence. In our study, genome-wide CNVs in 521 Chinese children with CAVC and SV were screened. A total of 27 CNVs ≥ 200 kb was detected, comprising 10 deletions and 17 duplications, in 11.52% (60/521) CHD cases, namely 16.79% (44/262) in CAVC cases and 6.18% (16/259) in SV cases. According to our strategy, 6 potentially-causative CNVs in 43 cases were identified and contributed to 16.41% (43/262) CAVC patients. Whereas, 6 potentially-causative CNVs in 6 cases were classified which led to the contribution to 2.32% (6/259) SV cases. CNVs in isolate/syndromic CHD patients have been investigated previously, providing a genome-wide (likely) pathogenic CNV burden ranging from 4.3 to 27.9% [52–55]. In our study, the rate of potentially-causative CNVs in SV cohort is relatively lower, possibly due to the subphenotype difference of CHD and/or the different stringency in variant interpretation standards. Based on previous study, different cardiac subphenotypes showed various enrichment of large CNV events [55], that is, the detection rates in various types of CHD were different. Additionally, some CNVs < 200 kb, which ignored in the present study, may be pathogenic. Furthermore, genome-wide CNVs with a minor allele frequency (MAF) < 1% are usually recognized as an important contributor to CHD [56] and majority non-causative CNVs in our study had a MAF < 1% in DGV database (Tables 2, 3). The conservative CNV analytic methods used in our study, including the restricted focus on CNVs that were absent in DGV, may result in missing some functional CNVs. Further study of these CNVs is still needed to evaluate the clinical implication.

## Conclusion

In conclusion, we identified 12 potentially-causative CNVs in 521 CAVC and SV patients, which represented the largest cohort of these two rare CHD types in China. Most CNVs identified in our study were rare (< 1%), occurring just once among the CAVC or SV samples except the 21q11.2–21q22.3 duplication in CAVC cohort. In this study, Chinese CAVC patients were mostly 21 trisomy with DS, which was consistent with the previous reports. Furthermore, it also suggested that there was no race difference in the close correlation between CAVC and DS patients. Combined with the present CNVs reports of CHD and the intolerance of genes within the CNVs regions, our results provided novel genetic evidences that could help clarify the etiology of CHD. Additionally, the potentially-causative CNVs we detected were seldom overlapped with known CHD loci, which implicated that abundant gene involved in heart development and diverse genetic causes of CHD.

## Supplementary Information

**Additional file 1**.** Table S1**. Phenotypes of CHD cohort.

## Data Availability

Data generated or analyzed during this study are included in this published article and its additional file. The raw data reported in this paper have been deposited in the OMIX, China National Center for Bioinformation/Beijing Institute of Genomics, Chinese Academy of Sciences (https://ngdc.cncb.ac.cn/omix:accession no.OMIX561). CNVs in our study were called based on human assembly GRCh38 (hg38, https://www.ncbi.nlm.nih.gov/assembly/GCA_000001405.27/).

## Abbreviations

CAVC: Complete Atrioventricular Canal
SV: Single Ventricle
ToF: Tetralogy of Fallot
ASD: Atrial septal defect
CoA: Coarctation of the Aorta
VSD: Ventricular Septal Defect
TGA: Transposition of Great Arteries
PFO: Patent foramen oval
PDA: Patent ductus arteriosus
